# The role of a starch-based diet in solving existential challenges for the 21st century

**DOI:** 10.3389/fnut.2023.1260455

**Published:** 2023-09-27

**Authors:** John McDougall

**Affiliations:** The McDougall Program, Santa Rosa, CA, United States

**Keywords:** diet, chronic disease, climate change, heart disease, COVID-19, greenhouse gas, animal agriculture, vegan

## Abstract

Diet plays a fundamental role in our major chronic diseases, in climate change, and in our resistance to infectious diseases. The eating patterns that best support the health of people and our planet are based on traditional starchy staples. Historically, a wide variety of starches have provided the bulk of the food for most of the people who have walked our Earth. For example, rice has been food for Asians, corn for Central Americans, potatoes for people of the Andes, and for the Middle East, “the bread basket of the world,” food has meant wheat and barley. Focusing on our ethnicities can expose altruistic natures, and before it is too late, allow us to *make the change* from destructive animal-food based-diets to plant-food based-diets; ones that are *health-supporting* for people and our planet.

## Introduction

Diet is the common denominator for mitigating the negative impacts of chronic diseases, climate changes, and now the COVID-19 pandemic. Each in its own right is threatening the lives of every human being on the planet. Progress in solving problems created by the first two challenges, chronic diseases and climate change, has been negligible; we seem content with eventual doom, barely noticing our demise, like the proverbial frog in the pot of water brought to a slow boil. However, truly radical behavior would be to let destructive dietary behaviors to continue, as they currently are, unchecked.

Fortunately, the novel coronavirus pandemic has acted as a high decibel alarm for these impending catastrophes; and as a “time machine” moving us a decade ahead in terms of focusing our attention on solving all three of these broad cultural calamities. Because of this viral pandemic, in fewer than 12 months, we have traded business- and pleasure-related travel for work- and leisure-experiences centered around the home, fossil fuels are being rapidly replaced by renewable energy sources, and our minds are now opening to once unthinkable possibilities for solving the world’s existential challenges. Although only a small step towards planetary recovery, a 10% reduction in greenhouse gas (GHG) has occurred within the first year of the first appearance of the first novel coronavirus, SARS-CoV2 (1).

## Chronic disease: it’s the food!

Worldwide and historically, chronic diseases, including obesity, diabetes, CHD (atherosclerosis), arthritis, kidney disease, and GI disorders are found exclusively in financially affluent social castes. Over 80% of people in developed countries worldwide now suffer from one or more of these medical conditions (2).

The cause is diet: classical paintings and writings portray the obese king with his gout inflicted foot propped on a stool, sitting with his sickly-looking royal court, before an opulent spread of animal foods (cows, pigs, chickens, and fishes); followed by pastries for the final culinary festivity.

Healing with diet-therapy for these chronic diseases has its roots in history also; and diet-therapy should be fundamental to the practice of medicine today, in the 21st century. Diet-therapy is the treatment of diseases by causing improvements in people’s short- and long-term food patterns. One of the earliest descriptions of this “low-tech medicine” is provided by a controlled trial carried out nearly 2,600 years ago, and reported in the first chapter of Daniel of the Bible (1:12–15): *Daniel’s men, who ate only vegetables (pulses) and drank water for a study period of 10 days were found to be stronger, healthier, younger-looking, more-handsome, and/or without-blemish, when compared to men eating at the king’s table.*

Historically, the wealthy class was limited to only a few members; the king and his personal court. At the other end of the spectrum were the masses, the working populations, who obtained the bulk of their calories from beans, corn, potatoes, rice, and/or wheat with a few fruits and vegetables. These people were strong, fit, and healthy (as long as they had enough food). With the Industrial Revolution and the harnessing of fossil fuels in the mid-1800s, wealth came to common folks; followed by a shift to very rich meals (the kind once reserved for the privileged few); and as expected, diseases of affluency quickly became epidemics, and now they are pandemic (worldwide) (3).

## Climate change: it’s the food!

I, along with most of you, was first made aware of the existential perils of climate change by Al Gore’s documentary, *An Inconvenient Truth,* in 2006. He clearly laid out the disastrous future ahead with his solutions being largely limited to curtailing the fossil fuel industries. No mention was made of the livestock industries’ contributions to global warming. Nor did his 2017 *Sequel* provide enlightenment on the potential role of curtailing our addiction to meat-eating for saving our home. In 2006, the Food and Agriculture Organization of the United Nations (FAO) published highly-influential research, *Livestock’s Long Shadow;* a nearly 400-page report finding livestock’s contribution to GHG production to be nearly 18% of the total; while all transportation (buses, trains, cars, planes, etc.) accounts to less than 14% (4). When accounting for several additional factors, those underestimated by the FAO report, livestock actually accounts for 51% of the GHG produced annually (5).

The *EAT*-*Lancet* Commission, considered to be the authoritative word on the subject of climate crises and diet, published their conclusions in 2019: The livestock industry is a major detriment to planetary survival; and that we have fewer than 12 years to take action before tipping points are reached where life as we once knew it will no longer be possible. In March of 2020, the *EAT-Lancet* Commission reported that replacing beef, pork, and poultry with a plant-food based-diet reduced the GHG associated with people’s diets by up to 50%, depending on the type and degree of substitution (vegan being most effective) (6). Further encouragement about the power of food comes from scientific findings that changing from a standard Western diet to a vegan diet can reduce GHG production by as much as 80% (7).

## COVID-19: it’s the food!

While people waited in line for an effective and safe vaccine to become available for them, they forfeited another powerful, cost-free, side-effect-free, self-administered intervention: the resolution of co-existing conditions, such as obesity, diabetes, hypertension, heart disease, and kidney disease. People, professionals, and politicians have largely ignored the fact that all of these conditions are due to the foods we eat—the rich Western diet—and these illnesses are easily cured by eating a starch-based diet (8, 9).

Anthony Fauci, MD from the NIH, told members of congress in a June 2020 meeting that, “…whether or not you have serious consequences, hospitalizations, intubations, and death, relate very strongly to the prevalence and incidence of underlying comorbid conditions…” (10). In other words, healthier people do not become mortally ill as often. This same optimistic prognosis was also made by the Centers for Disease Control and Prevention (CDC) on June 15, 2020 when the Morbidity and Mortality Weekly Report (MMWR) announced the risk of hospitalization is increased 6-fold and risk of death by 12-fold in people with co-existing diseases (11). Overseas, the editor of the *British Medical Journal*, July 20, 2020, reminded readers that in order to deal effectively with this pandemic, “we need diets richer in whole grains, fruit, vegetables, and legumes, with less red meat, refined carbohydrates, and highly processed foods” (12).

Vaccinations have brought hope to people—an expectation that someday this nightmare will be over. Unfortunately, variants are emerging worldwide. The problem being with the genetic drift common to viruses, fueled by a warming planet, a plethora of novel contagious microbes will become our future. Does this mean a new vaccine every 3 months? Possibly. Let us not forget that we have highly effective public health measures (masks, social distancing, hand-washing), and the widespread adoption of traditional diets that eliminate comorbid- or premorbid- conditions, almost overnight.

## Back to the future with traditional diets

A simple U-turn, back to traditional diets followed by the more than 107 billion human inhabitants who have walked planet Earth, is the change in eating patterns I am proposing. All large populations of trim, healthy, athletic-competing, war-fighting people throughout verifiable human history have obtained the bulk of their calories from starches. Examples of thriving populations include the Japanese, Chinese, and other Asians, who ate sweet potatoes, buckwheat, and/or rice; Incas in South America who ate potatoes; Mayans and Aztecs in Central America were known “as the people of the corn;” and the Middle East, formally known as “the breadbasket of the world,” fed hundreds of millions on a diet of wheat and barley. I say “ate,” rather than “eat,” because, since the early 1980s Western eating patterns have prevailed over traditional eating patterns based on staples, like corn, potatoes, rice, and wheat. This is, by no coincidence, the period of time that the greatest damage to Earth from climate change has occurred.

The vast majority want a viable future for their children; they just do not know how to attain this elusive, but precious, goal. People are poised for big changes in their lives, especially after realizing that there is no going back to “normal,” and that in fewer than 30 years, nearly one-third of all plants and animals are predicted to become extinct (13, 14). Some may believe that, “More research and more discussion need to be done before we act.” We do not have the luxury of time to make our conclusions acceptable to everyone. Present circumstances dictate that observations, deductive reasoning, incomplete science, and a bit of faith must prevail in our decision to act now.

Looking back, to only 50 years ago, the majority of people on Earth followed a starch-based diet; and many of us are old enough to remember these times. How ironic to realize how knowledge commonly held within one lifetime—the traditional starch-based diet—would also be a necessary part of the solution to mitigating climate change for the next half century, and beyond, for the lifetimes of many future generations. We have a world to save and every bite counts.

## Data availability statement

The original contributions presented in the study are included in the article/supplementary material, further inquiries can be directed to the corresponding author.
